# Acute MDMA and Nicotine Co-administration: Behavioral Effects and Oxidative Stress Processes in Mice

**DOI:** 10.3389/fnbeh.2018.00149

**Published:** 2018-08-02

**Authors:** Barbara Budzynska, Artur Wnorowski, Katarzyna Kaszubska, Grazyna Biala, Marta Kruk-Słomka, Jacek Kurzepa, Anna Boguszewska-Czubara

**Affiliations:** ^1^Department of Pharmacology and Pharmacodynamics, Medical University of Lublin, Lublin, Poland; ^2^Department of Biopharmacy, Medical University of Lublin, Lublin, Poland; ^3^Department of Medical Chemistry, Medical University of Lublin, Lublin, Poland

**Keywords:** MDMA, nicotine, memory, behavioral sensitization, oxidative stress, cholinergic receptors

## Abstract

3,4-Methylenedioxy-methylamphetamine (MDMA), a synthetic substance commonly known as ecstasy, is a worldwide recreational drug of abuse. As MDMA and nicotine activate the same neuronal pathways, we examined the influence of co-administration of nicotine (0.05 mg/kg) and MDMA (1 mg/kg) on cognitive processes, nicotine-induced behavioral sensitization and on processes linked with oxidative stress and α7 nicotinic acetylcholine receptors (nAChRs) expression in the brain of male Swiss mice. For behavioral study the passive avoidance (PA) test and locomotor sensitization paradigm were used. Also, the oxidative stress parameters as well as expression levels of α7 nAChRs in prefrontal cortex and hippocampus of mice treated with MDMA alone or in combination with nicotine were assessed. The results revealed that MDMA injections as well as co-administrations of MDMA and nicotine improved memory consolidation in male Swiss mice tested in PA task. Furthermore, one of the main findings of the present study is that MDMA increased locomotor activity in nicotine-sensitized mice. Our study showed for the first time strong behavioral and biochemical interactions between nicotine and MDMA. Both drugs are very often used in combination, especially by young people, thus these results may help explaining why psychoactive substances are being co-abused and why this polydrug administration is still a social problem.

## Introduction

3,4-Methylenedioxy-methylamphetamine (MDMA), a synthetic substance well known as ecstasy, is a worldwide recreational drug of abuse. Such abuse results in fatal cases especially among young people. MDMA is a stimulant of the central nervous system (CNS) possesses hallucinogenic properties described as an *increased sensory awareness* (Morton, 2005; George et al., 2010). It also induces neurotoxicity (Gonçalves et al., 2014). Illicit MDMA is typically manufactured in a form of tablets of varying purity, with ketamine, amphetamine and caffeine being the most common contaminants (Tanner-Smith, 2006). A significant issue related to the MDMA abuse is the co-administration of MDMA with other stimulants: ethanol, amphetamines, cocaine, cannabis or nicotine (UNODC World Drug Report. In fact, abuse of MDMA by tobacco smokers has been commonly reported, especially in a nightclub setting (Mohamed et al., 2011; UNODC World Drug Report http://www.unodc.org/unodc/en/data-and-analysis/WDR-2010.html, 2010)^1^. Hence, nicotine present in tobacco may modulate the behavioral and neurochemical effects of MDMA in real-life situations.

Although the acute and chronic effects of MDMA in animal models have been widely described (Navarro and Maldonado, 2002; Cole and Sumnall, 2007; Viñals et al., 2013), very few studies have evaluated the consequences of polydrug associations. Data on behavioral effects of concomitant administration of MDMA and nicotine are also scarce. It is well established that MDMA by itself reduced the activity of serotonin (5-HT) transporters in the brain. This process is supposed to be a presumable mechanism contributing to 5-HT release, the main pharmacological effect of MDMA. MDMA is able to elevate dopamine (DA) and noradrenaline (NA) levels, although this effect is less pronounced. MDMA also delays metabolism by inhibiting monoamine oxidase (MAO) and binds to distinct 5-HT and NA receptors (Leonardi and Azmitia, 1994; White et al., 1996; Capela et al., 2009). Aforementioned mechanisms lead to the extracellular increase of neurotransmitters level in CNS (for review see Budzynska et al., 2017). Moreover, MDMA has affinity for neuronal nicotinic acetylcholine receptors (nAChRs) and acts as a partial agonist of alpha7 nAChR (Chipana et al., 2008; Garcia-Ratés et al., 2010). nACHRs are highly distributed in the CNS and promote the release of not only acetylcholine (ACh) but also DA, NA, 5-HT and gamma-aminobutiric acid (GABA) (Wonnacott et al., 2005). It is well documented that nicotine contained in tobacco acts through nAChRs. Nicotinic receptors play a pivotal role in nicotine addiction, sensitization as well as in other behavioral effects such as cognitive effects and anxiety-like behavior (File et al., 2000), analgesia (Marubio et al., 1999), or depressive-like behaviors (Hayase, 2011). Additionally, MDMA behaves as a partial agonist of α7 nAChR, and as an antagonist on α4β2 nAChR (Garcia-Ratés et al., 2010), whereas nicotine activates both α7 and α4β2 types of nAChRs in the brain. However, central effects of common intake of MDMA and nicotine still remains undiscovered.

The exact mechanism underlying MDMA multiple organ toxicity is not well understood. We hypothesize that MDMA-induced toxicity results from a loss of mitochondrial functions, overproduction of reactive oxygen species (ROS) and arising oxidative stress (Gonçalves et al., 2014). In such a scenario, an organism activates a protective antioxidant barrier to prevent severe oxidative damage. This barrier consists of a set of antioxidant enzymes and several types of small molecules that act as free radical scavengers (Kruk-Slomka et al., 2016); however, MDMA may disrupt the barrier through down modulation of the enzymes and depletion of the scavengers.

The combining of various psychoactive substances is common for addicted people. However, the explanation of drug or poly-drug addiction cannot be only the psychoactive properties of the drugs (Hyman, 2005). Memory and learning processes may also underlie these effects (Hyman et al., 2006). Therefore, the aim of the present behavioral experiments was: (1) to evaluate the influence of co-administration of MDMA and nicotine on memory consolidation processes and (2) to investigate the effect of MDMA on the expression of nicotine-induced behavioral sensitization. In parallel, biochemical studies included determinations of the antioxidant enzymes activities: superoxide dismutase (SOD), glutathione reductase (GR) glutathione peroxidase (GPx), and concentration of malondialdehyde (MDA), within the hippocampus, the prefrontal cortex and the whole brain. Moreover, expression of α7 nACh receptors in prefrontal cortex and hippocampus upon MDMA and nicotine treatment was determined.

## Methods

### Animals

Behavioral studies were carried out on 6 week old male Swiss mice (Farm of Laboratory Animals, Warsaw, Poland) weighing 20-25 g at the beginning of the experiments. The animals were kept under standard laboratory conditions (room temperature 21 ± 1°C, 12 h light/dark cycle,) with free access to laboratory chow and tap water (Agropol, Pulawy, Poland) and were become adjusted to new conditions in the laboratory for at least 1 week. Each experimental group consisted of 8-10 animals. All experiments/procedures were organized and carried out in agreement with the National Institute of Health Guidelines for the Care and Use of Laboratory Animals and to the European Community Council Directive for the Care and Use of Laboratory Animals of 22 September 2010 (2010/63/EU) and were approved by the 1st Local Ethics Committee for Animal Experiments in Lublin, Racławickie 1 street (agreement no. 15/2012).

### Drugs

The range of doses of nicotine and MDMA were chosen based on literature data (Ciudad-Roberts et al., 2013), our recently published articles (Biala and Staniak, 2010; Budzynska et al., 2017) and preliminary studies. Our previous experiments showed that administration of nicotine at the dose of 0.05 mg/kg had no effect on memory consolidation (Budzynska et al., 2013). Whereas, the higher dose of nicotine (0.5 mg/kg) enhanced cognitive processes in the animals. Also repeated intermittent administration of nicotine (0.5 mg/kg) induce locomotor sensitization (Biala and Staniak, 2010). The dose of MDMA in the PA paradigm was chosen on the basis of our previous study (Budzynska et al., 2017) as inactive in this test.

### Chemicals

Cell Lysis Buffer was obtained from Cell Signaling Technology (Danvers, MA, USA). Halt protease and phosphatase inhibitor cocktail was from Thermo Fisher Scientific (Waltham, MA, USA) whereas phenylmethanesulfonyl fluoride (PMSF) was from Sigma-Aldrich. The bicinchoninic acid (BCA) assay kit, 4-12% pre-cast polyacrylamide gels, transfer stacks with polyvinylidene fluoride (PVDF) membranes, and Pierce peroxidase substrate for enhanced chemiluminescence (ECL) were all purchased from Thermo Fisher Scientific.

### Passive avoidance test

The passive avoidance apparatus and procedure was described in detail in our previous article (Budzynska et al., 2015). The apparatus consisted of two-chamber box with a lighted and darkened compartment. The light part was illuminated by a fluorescent light (8 W) and was connected to the dark chamber which was equipped to with an electric grid floor. Entrance of the animals to the dark box was punished by an electric shock to the feet (0.15 mA for 2 s) (Javadi-Paydar et al., 2012).

### Locomotor activity

Locomotor activity was measured with photoresistor actimeters (circular cages, diameter 25 cm, two light beams). Mice were individually placed in an actimeter for 60 min. The number of times the mice crossed the light beams was recorded as the locomotor activity after 60 min for evaluation effect of MDMA on locomotor activity in nicotine-sensitized mice. Whereas, spontaneous locomotor activity was recorded after 20, 40 and 60 min after MDMA injections.

### Experimental procedure and treatment

#### Experiment 1: effect of MDMA on spontaneous locomotor activity

For evaluation of locomotor effects of MDMA (0.1, 0.5, 1, 5, 10, and 20 mg/kg) the animals, were injected with MDMA or saline for the control group, and immediately placed in the activity chamber. Locomotor activity, i.e., the number of photocell beam breaks was automatically recorded for 60 min.

#### Experiment 2: effects of MDMA alone and co-administration of MDMA and nicotine on memory related behaviors in the PA Test

On the first day of training (pre-test), mice were placed individually into the light compartment/chamber and allowed to explore the light box/ compartment. After 30 s, the guillotine door was raised to allow the mice enter the dark compartment/ chamber/box. When the mice entered the dark compartment, the guillotine door were closed and an electric foot-shock (0.15 mA) of 2 s duration was delivered immediately to the animal. The latency time for entering the dark compartment was recorded (TL1).

In the retention trial 24 h later, the same mouse was a second time placed individually in the light chamber of the PA apparatus and the time taken to reenter the dark compartment was recorded (TL2). No foot-shock was applied in retention (Javadi-Paydar et al., 2012). The experimental procedure involved examination of memory consolidation (the animals received injections of the substance after pre-test to avoid the psychostimulant effect that could alter the performance in the PA paradigm). During the acute treatment, the animals were allocated to the following drug groups: vehicle + vehicle, vehicle + nicotine (0.05 mg/kg, s.c.), vehicle + MDMA (1 mg/kg, i.p.), or MDMA (1 mg/kg, i.p.) co-administered with nicotine (0.05 mg/kg, s.c.). The drugs were administered immediately after pre-test (memory consolidation), and the mice were re-tested 24 h later.

#### Experiment 3: effect of MDMA on locomotor activity in nicotine-sensitized mice

This method was similar to that used in our previous experiments according to the data indicating that the dose of 0.5 mg/kg of nicotine produces robust locomotor sensitization in mice under our laboratory conditions (Biala and Staniak, 2010). During the pairing phase (days 1-9), mice received the following injections: saline (i.p.) + saline (s.c.) or saline (i.p.) + nicotine (0.5 mg/kg, s.c.) every other day for five sessions. The mice remained drug free for 1 week and, on day 16, the same groups of mice were further challenged with nicotine (0.5 mg/kg, s.c.), or saline, respectively. On the challenge day (day 16) the mice pretreated with saline or nicotine (as mentioned above) were injected with MDMA (5 mg/kg). Locomotor activity was measured for 60 min during the pairing phase (days 1-9) and on the 16th day, immediately after injections. We have chosen the dose of MDMA (5 mg/kg) not influencing the locomotor activity administered alone.

### Biochemical determinations

#### Collection of tissues

The animals after the behavioral tests were sacrificed by decapitation and their brains were collected, rinsed in ice-cold saline and then the structures (the cerebrum, the cerebral cortex and the hippocampus) were immediately separated. The isolated structures as well as whole brain were used for the experiments.

#### Preparation of brain homogenates

To obtain homogenates the tissues were homogenized in ice-cold 0.1 M Tris buffer (pH 7.4) and centrifuged to separate nuclear debris. Collected supernatant was used for the spectrophotometrical determination of TAS, activity of SOD and GPx, concentration of MDA and protein level on HITACHI 2800 apparatus and microplate reader EPOCH.

#### Determination of malondialdehyde concentration (MDA)

The concentration of malondialdehyde was determined to analyze the level of lipid peroxidation. Thiobarbituric acid (TBA) reaction (Ledwozyw et al., 1986) was used as described in Budzynska et al. (2015). The concentration of MDA was expressed as μM of MDA/g of wet tissue.

#### Determination of total antioxidant status (TAS)

To determine TAS in the homogenates ready-to-use diagnostic kit TAS by RANDOX (Randox Laboratories Ltd., UK) was used according to manual. The low molecular weight antioxidants concentration in the tissues homogenates were determined on HITACHI 2800 spectrophotometer by absorption measurements at 600 nm (Biala et al., 2017). Results are expressed in μM/g tissue.

#### Determination of superoxide dismutase activity (SOD) and glutathione peroxidase activity (GPx)

The activities of SOD and GPx was measured with the use of ready-to-use diagnostic kits RANSOD and RANSEL (by Randox), respectively. The determination of SOD employs the generation of superoxide radicals in the reaction of xantine and xantine oxidase (XOD) with iodonitrotetrazolium chloride to form red formazan dye (Supplementary Datasheet1). The increase in absorbance at 505 nm is read to calculate the superoxide dismutase activity from standard curve. The determination of GPx activity is based on a method of Paglia and Valentine (Paglia and Valentine, 1967). The absorbance is measured at 340 nm. Results of both enzymes activity are expressed in U/g protein.

#### Determination of protein content

The concentration of proteins in tissues homogenates was measured with the use of Pierce BCA Protein Assay Kit by Thermo Scientific.

### Statistical analysis

The data were expressed as the mean ± standard error of the mean (SEM). The statistical analyses were performed by the two-way or one-way analysis of variance (ANOVA). Post hoc comparison of means was carried out with the Tukey's test for multiple comparisons, when appropriate. The confidence limit of *p* < 0.05 was considered statistically significant.

For the memory related behaviors, changes in the PA performance were expressed as the difference between retention and training latencies and were taken as an index of latency (IL). IL was calculated for each animal and reports as the ratio:

$$\begin{array}{l} \text{IL}=\text{TL}2-\text{TL}1/\text{TL}1 \end{array}$$

TL1-the time taken to enter the dark compartment during the training, TL2-the time taken to reenter the dark compartment during the retention.

## Results

### Influence of MDMA on locomotor activity

**Figure 1:**
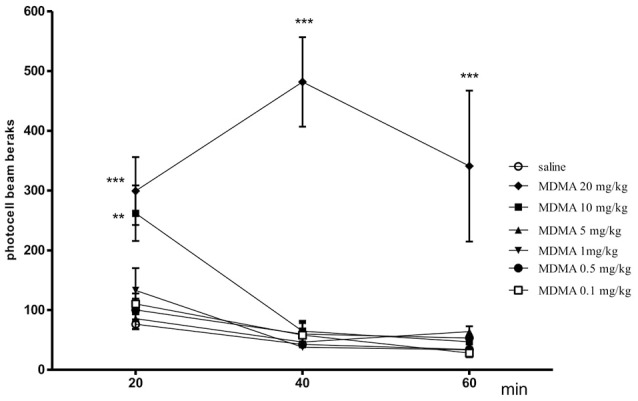
Effect of MDMA on spontaneous locomotor activity in mice. MDMA (0.1, 0.5, 1, 5, 10, and 20 mg/kg, i.p.) or saline were administered immediately before the test; *n* = 8 − 10; Data represent the means ± SEM; **p < 0.01; ***p < 0.001; vs. saline control group; Tukey's test.

The effect of MDMA on locomotor activity in mice after 20 min [one-way ANOVA *F*_(6, 50)_ = 7.7450, *p* = 0.0001], 40 min [one-way ANOVA *F*_(6, 50)_ = 17.510, *p* = 0.0001] and 60 min [one-way ANOVA *F*_(6, 50)_ = 13.720, *p* = 0.0001] is shown in Figure 1. The post hoc Tukey's test revealed that MDMA administered at the doses of 20 and 10 mg/kg significantly increased locomotor activity after 20 and 40 min (*p* < 0.001, *p* < 0.01, respectively) as well as 40 and 60 min (20 mg/kg-*p* < 0.001) as compared with the saline-treated mice. Moreover, MDMA (0.1, 0.5, 1, and 5 mg/kg) given acutely did not caused changes in the locomotor activity of animals.

### Effects of co-administration MDMA and nicotine on cognitive-like behaviors observed in the PA test

**Figure 2:**
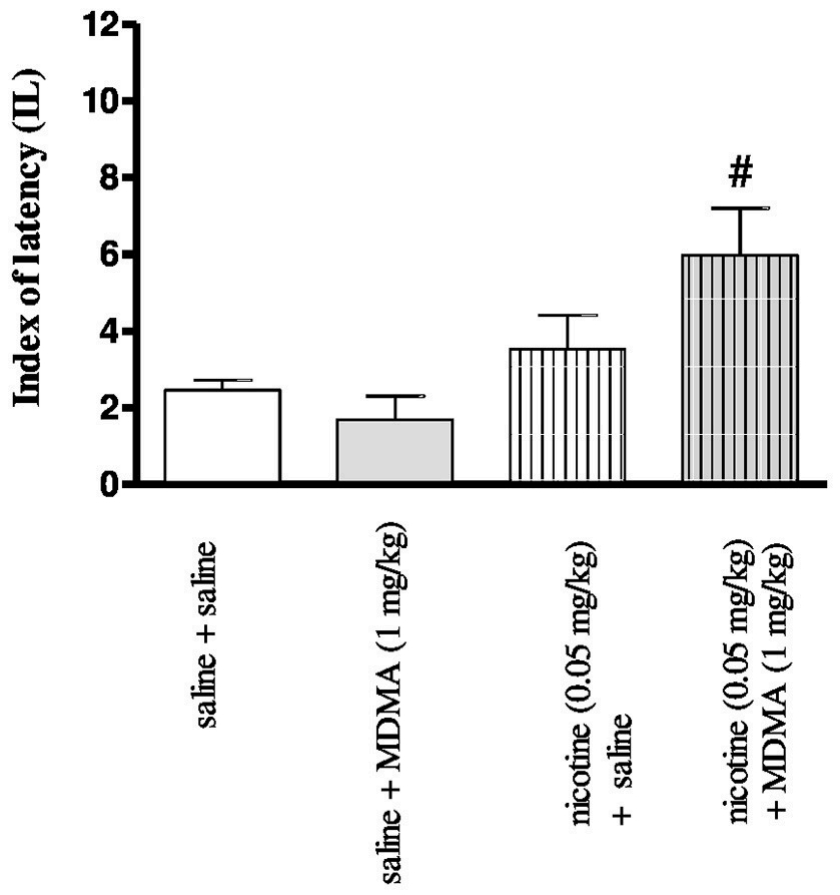
Effects of co-administration of MDMA (1 mg/kg, i.p.) and nicotine (0.05 mg/kg, s.c.) on the memory consolidation trial using the PA test in mice. Appropriate groups of mice received compounds immediately after the pre-test. Data represent the means ± SEM and are expressed as latency index (IL); *n* = 8 - 10; #*p* < 0.05; vs. MDMA-treated control group; Tukey's test.

Figure 2 depicts the effects of injection of nicotine (0.05 mg/kg, s.c.) and MDMA (1 mg/kg, i.p.) in combination on memory consolidation during the retention trial in the PA task [two-way ANOVA: pre-treatment *F*_(1, 30)_ = 85.00, *p* < 0.001, treatment *F*_(1, 30)_ = 8.33, *p* = 0.0072, and interactions *F*_(1, 30)_ = 30.45, *p* < 0.001]. The nonactive, in PA test, doses of MDMA (1 mg/kg) and nicotine (0.05 mg/kg) were chosen for studies of the interactions of both drugs in cognitive processes (Budzynska et al., 2013). Statistically significant amelioration of cognitive function was observed in the animals administered with nicotine (0.05 mg/kg) and MDMA (1 mg/kg) in combination *vs*. the MDMA-treated mice (*p* < 0.05).

### The evaluation of effect of MDMA on locomotor activity in nicotine-sensitized mice

**Figure 3:**
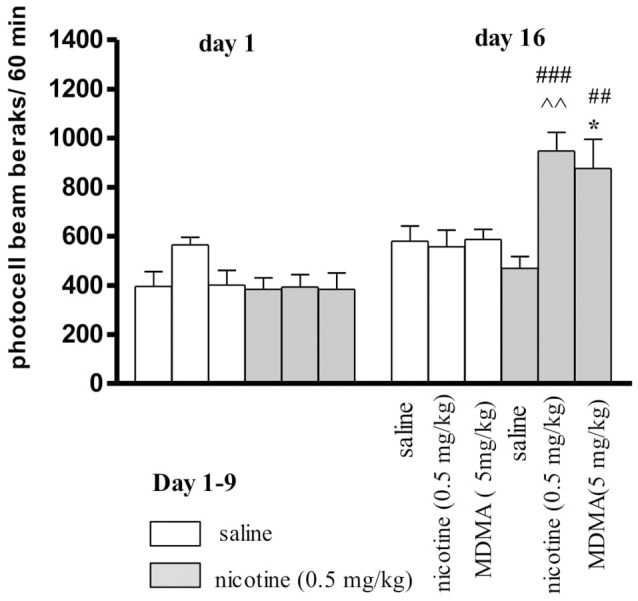
Effects MDMA (5 mg/kg, i.p.) on the expression of locomotor sensitization to nicotine in mice. Nicotine (0.5 mg/kg, s.c.) or saline were injected daily for 5 days, every other day; on day 16 (a test for expression of sensitization) mice were given nicotine (0.5 mg/kg), saline, or MDMA (5 mg/kg). Data represent means ± SEM; *n* = 8 - 10 mice per group. ###*p* < 0.001, ##*p* < 0.01 vs. the first pairing day; ‘*p* < 0.01 *vs*. saline-pretreated and nicotine-challenged mice; ^*^p < 0.05 vs. saline-pretreated and MDMA-challenged mice (Tukey's test).

It was revealed that locomotor response after administration of nicotine (0.5 mg/kg, s.c.) or saline during the pairing phase (day 1 and day 16-challenge) exert statistically significant treatment effect [*F*_(5, 69)_ = 4.18, *p* < 0.0165], a day effect [*F*_(1, 69)_ = 26.87, *p* < 0.0001] and an interaction effect [*F*_(5, 69)_ = 11.12, *p* < 0.0001] (Figure 3). On the 1st day of experiment, no significant treatment effect was observed [*F*_(1, 48)_ = 6.543, *p* = 0.1785]. On the last (16th) day, after an additional injection of nicotine, a significant treatment effect was observed [*F*_(5, 48)_ = 3.988, *p* = 0.0046]. The challenge nicotine injection (day 16), induced a significant effect observed as difference between the response to the first injection of nicotine (*p* < 0.001) or to nicotine in animals treated with repeated saline (*p* < 0.01, Tukey's test) (Figure 3). Moreover, MDMA, at the dose of 5 mg/kg injected on the day 16 to the nicotine-pretreated group significantly increased the locomotor activity of the mice as compared to the first injection of nicotine (*p* < 0.01) and to the group of animals repeatedly treated with saline (*p* < 0.05, Tukey's test)

### The effect of MDMA on oxidative stress indicators

The effect of MDMA on oxidative stress indicators (TAS, SOD, GPx, and MDA) in the whole brain as well as in separated the hippocampus and the cortex of mice receiving saline or four different doses of MDMA (1, 5, 10, 20 mg/kg) are presented in Table 1. Performed statistical analysis revealed that MDMA administration caused statistically significant changes in TAS value within the cortex [one-way ANOVA *F*_(4, 25)_ = 2.997, *p* = 0.0378] and the hippocampus [one-way ANOVA *F*_(4, 25)_ = 4.827, *p* = 0.0050], in SOD [one-way ANOVA *F*_(4, 41)_ = 4.231, *p* = 0.0059] and MDA [one-way ANOVA *F*_(4, 41)_ = 8.725, *p* < 0.0001] within the whole brain.

**Table 1:** The effect of MDMA on oxidative stress indicators (TAS, SOD, GPx, and MDA) in whole brain as well as in separated the hippocampus and the cortex of mice receiving saline or four different doses of MDMA (1, 5, 10, 20 mg/kg b.w.).

**Oxidative stress parameter**	**Structure**	**Saline**	**MDMA 1 mg/kg**	**MDMA 5 mg/kg**	**MDMA 10 mg/kg**	**MDMA 20 mg/kg**
TAS	Brain *F*_(4, 25)_ = 2.031 *P* = 0.1207	294.6 ± 109.4	293.6 ± 69.56	232.2 ± 62.85	232.9 ± 59.22	202.7 ± 23.99
	Cortex *F*_(4, 25)_ = 2.997 *P* = 0.0378	266.3 ± 34.72	265.6 ± 38.73	237.3 ± 35.05	214.4 ± 22.56	202.1 ± 29.03
	Hippocampus *F*_(4, 25)_ = 4.827 *P* = 0.0050	387.3 ± 43.79	404.6 ± 33.98	380.2 ± 69.78	336.1 ± 42.57	320.7 ± 54.72^*^
SOD	Brain *F*_(4, 41)_ = 4.231 *P* = 0.0059	3.04 ± 0.45	2.98 ± 0.67	2.67 ± 0.56	2.56 ± 0.36	2.21 ± 0.49^**^
	Cortex *F*_(4, 25)_ = 0.2933 *P* = 0.8795	14.50 ± 3.00	11.80 ± 2.79	12.47 ± 1.77	10.18 ± 2.01	9.880 ± 1.70
	Hippocampus *F*_(4, 25)_ = 3.933 *P* = 0.0131	17.63 ± 2.61	17.44 ± 2.36	17.14 ± 4.66	16.84 ± 2.88^*^	15.84 ± 2.91^*^
GPx	Brain *F*_(4, 25)_ = 1.085 *P* = 0.3851	16.42 ± 3.39	17.50 ± 3.74	15.39 ± 2.45	14.90 ± 2.58	14.31 ± 2.53
	Cortex *F*_(4, 25)_ = 1.069 *P* = 0.3925	70.30 ± 11.82	75.96 ± 14.04	69.14 ± 8.80	67.32 ± 7.10	64.03 ± 8.67
	Hippocampus *F*_(4, 25)_ = 2.382 *P* = 0.0786	88.49 ± 13.02	83.97 ± 11.58	85.60 ± 12.61	76.31 ± 13.61	69.25 ± 11.24
MDA	Brain *F*_(4, 41)_ = 8.725 *P* < 0.0001	24.04 ± 5.14	26.43 ± 4.39	31.24 ± 4.33^*^	31.52 ± 6.94^*^	37.28 ± 5.06^***^
	Cortex *F*_(4, 25)_ = 3.475 *P* = 0.0218	13.15 ± 1.77	14.20 ± 1.88	15.13 ± 1.76	16.58 ± 2.61	18.00 ± 3.90
	Hippocampus *F*_(4, 25)_ = 2.061 *P* = 0.1163	2.620 ± 0.71	2.458 ± 0.69	2.828 ± 0.70	3.062 ± 0.52	3.467 ± 0.73^*^

*Data represent means ± SD, n = 8-12 mice per group*;

* *p < 0.01*,

** *p < 0.005*,

*** *p < 0.001 vs. saline treated group (Tukey's test)*.

The post hoc Tukey's test revealed that increasing doses of MDMA caused decrease in the values of TAC in the whole brain as well as in the studied structures, however statistically significant decrease was noted only in case of dose of 20 mg/kg in the hippocampus (*p* < 0.01). Our experiment showed decrease in the activity of antioxidant enzymes (SOD and GPx) in applied MDMA dose. Statistically significant changes were noted in SOD activity in the whole brain at the highest MDMA dose (20 mg/kg, *p* < 0.005) and in the hippocampus in case of two highest doses (10 mg/kg, *p* < 0.01 and 20 mg/kg, *p* < 0.01). Our experiment proved that MDMA extended lipids peroxidation, what was measured as increase in MDA concentration. Statistically significant changes were stated in the whole brain at the doses of MDMA of 5 mg/kg (*p* < 0.01), 10 mg/kg (*p* < 0.01) and 20 mg/kg (*p* < 0.001) and in the hippocampus at the highest MDMA dose (20 mg/kg, *p* < 0.01).

### Effects of co-administration of MDMA and nicotine on oxidative stress indicators

The effect of co-administration of MDMA (1 mg/kg) and nicotine (0.05 mg/kg) on oxidative stress indicators (TAS, SOD, GPx, and MDA) in the whole brain as well as in separated the hippocampus and the cortex of mice are presented in Table 2.

**Table 2:** The effect of co-administration of MDMA (1 mg/kg b.w.) and nicotine (0.05 mg/kg b.w.) on oxidative stress indicators (TAS, SOD, GPx, and MDA) in whole brain as well as in separated hippocampus and the cortex of mice.

**Oxidative stress parameter**	**Structure**	**Saline**	**Nicotine**	**MDMA 1 mg/kg**	**MDMA 1 mg/kg + nicotine**
TAS	Brain	294.6 ± 109.4	255.0 ± 38.24	293.6 ± 69.56	246.2 ± 43.29^**^^#^
	Cortex	266.3 ± 34.72	246.0 ± 51.02	265.6 ± 38.73	225.0 ± 27.98
	Hippocampus	387.3 ± 43.79	373.9 ± 51.38	404.6 ± 33.98	358.1 ± 45.69
SOD	Brain	3.04 ± 0.45	2.672 ± 0.70	2.98 ± 0.67	2.420 ± 0.46
	Cortex	14.50 ± 3.00	11.11 ± 1.65^*^	11.80 ± 2.79	10.26 ± 1.34^**^
	Hippocampus	17.63 ± 2.61	14.64 ± 2.92	17.44 ± 2.36	14.62 ± 2.42
GPx	Brain	16.42 ± 3.39	15.44 ± 1.40	17.50 ± 3.74	15.12 ± 1.76
	Cortex	70.30 ± 11.82	65.10 ± 6.30	75.96 ± 14.04	61.21 ± 6.66^#^
	Hippocampus	88.49 ± 13.02	76.57 ± 12.45	83.97 ± 11.58	76.07 ± 12.03
MDA	Brain	24.04 ± 5.14	28.09 ± 6.95	26.43 ± 4.39	29.55 ± 7.21
	Cortex	13.15 ± 1.77	15.26 ± 1.99	14.20 ± 1.88	17.22 ± 2.99
	Hippocampus	2.620 ± 0.71	2.437 ± 0.61	2.458 ± 0.69	2.552 ± 0.47^**^^#^

*Data represent means ± SD, n = 8-12 mice per group*;

* *p < 0.01*,

** *p < 0.005 vs. saline treated control group*;

# *p < 0.01 vs. saline treated MDMA group (Tukey's test)*.

Two-way ANOVA of TAS value after administration of MDMA (1 mg/kg) with nicotine (0.05 mg/kg, s.c.) or saline revealed a pretreatment effect [*F*_(1, 36)_ = 4.600; *p* = 0.0388] without treatment [*F*_(1, 36)_ = 0.002885; *p* = 0.9575] and interaction effects [*F*_(1, 36)_ = 1.405; *p* = 0.2437] in the hippocampus and pretreatment effect [*F*_(1, 36)_ = 6.089; *p* = 0.0185] without treatment [*F*_(1, 36)_ = 0.7730; *p* = 0.3851] and interaction effects [*F*_(1, 36)_ = 0.6765; *p* = 0.4162] in the cortex, while no effects were stated in the whole brain (pretreatment [*F*_(1, 36)_ = 3.758; *p* = 0.0604]; treatment [*F*_(1, 36)_ = 0.04768; *p* = 0.8284]; interaction [*F*_(1, 36)_ = 0.03020; *p* = 0.8630].

The post hoc Tukey's test revealed that co-administration of MDMA with nicotine caused decrease in TAS value in the whole brain and separated structures, which was statistically significant in comparison to saline control group (*p* < 0.05) and MDMA saline treated group (*p* < 0.01) only in the whole brain.

Two way ANOVA of SOD activity revealed a pretreatment effect [*F*_(1, 36)_ = 5.217; *p* = 0.0284] in the whole brain without treatment [*F*_(1, 36)_ = 0.3181; *p* = 0.5762] and interaction effects [*F*_(1, 36)_ = 0.7110; *p* = 0.4047], pretreatment effect in the hippocampus [*F*_(1, 36)_ = 12.61; *p* = 0.0011] without treatment [*F*_(1, 36)_ = 0.01647; *p* = 0.8986] and interaction effects [*F*_(1, 36)_ = 0.01080; *p* = 0.9178] and pretreatment [*F*_(1, 36)_ = 11.42; *p* = 0.0018] and treatment effects in the cortex [*F*_(1, 36)_ = 5.923; *p* = 0.0200] without interaction effect [*F*_(1, 36)_ = 1.609; *p* = 0.2128].

The post hoc Tukey's test revealed that nicotine administered alone caused significant decreases in SOD activity in the cortex (*p* < 0.01), while co-administration of MDMA with nicotine caused decrease in SOD activity in the whole brain and separated structures, which was statistically significant (*p* < 0.05) in comparison to saline control group only in the prefrontal cortex.

Two way ANOVA analysis of GPx activity revealed no effects in the whole brain [pretreatment *F*_(1, 36)_ = 3.705; *p* = 0.0622; treatment *F*_(1, 36)_ = 0.1896; *p* = 0.6659; interaction *F*_(1, 36)_ = 0.6432; *p* = 0.4278], while in the hippocampus only pretreatment effect [*F*_(1, 36)_ = 6.511; *p* = 0.0151] without treatment [*F*_(1, 36)_ = 0.4177; *p* = 0.5222] and interaction effects [*F*_(1, 36)_ = 0.2678; *p* = 0.6079] and pretreatment effect in the cortex [*F*_(1, 36)_ = 9.457; *p* = 0.0040] without treatment [*F*_(1, 36)_ = 0.07444; *p* = 0.7865] and interaction [*F*_(1, 36)_ = 2.167; *p* = 0.1497].

The post hoc Tukey's test revealed that co-administration of MDMA with nicotine caused decrease in GPx activity, which was statistically significant in comparison to MDMA saline treated group (*p* < 0.01) only in the cortex.

The results of two-way ANOVA analysis of MDA concentration in the whole brain and the hippocampus did not reveal any effects (brain: pretreatment [*F*_(1, 36)_ = 3.525; *p* = 0.0686]; treatment [*F*_(1, 36)_ = 1.016; *p* = 0.3201]; interaction [*F*_(1, 36)_ = 0.05931; *p* = 0.8090] and the hippocampus: pretreatment [*F*_(1, 36)_ = 0.05029; *p* = 0.8238]; treatment [*F*_(1, 36)_ = 0.01402; *p* = 0.9064]; interaction [*F*_(1, 36)_ = 0.4872; *p* = 0.4897] while in case ofthe cortex pretreatment [*F*_(1, 36)_ = 13.46; *p* = 0.0008] and treatment effects [*F*_(1, 36)_ = 4.634; *p* = 0.0381] were stated without interaction effect [*F*_(1, 36)_ = 0.4236; *p* = 0.5193].

The post hoc Tukey's test revealed that co-administration of MDMA with nicotine caused increase in MDA concentration in the whole brain and separated structures: the hippocampus and the cortex. Statistically significant changes were stated only in the hippocampus in comparison to saline control group (*p* < 0.05) and MDMA treated group (*p* < 0.01).

## Discussion

The first set of experiments showed that co-administrations of MDMA and nicotine improved memory consolidation in male Swiss mice tested in the PA task. It is well established that the cholinergic neurotransmission plays a pivotal function in the cognitive process and thus physiological concentration of ACh is very important for proper brain function in both human and rodents. (Knoppman, 2001). It was established that different subtypes of nicotinic receptors are engaged in memory and learning processes. It was revealed that agonists of both α4β2 (ABT-418, RJR 2403) (Levin, 2002), and α7 nAChR (Anabaseine, ARR 17779) (Arendash et al., 1995) exert procognitive effect. Previous studies suggested that the effect of MDMA on memory and learning processes is dependent on the exposure regime. Memory impairment following pre-training administration of MDMA has been shown in adolescent mice in the PA task (Daza-Losada et al., 2009). Also, amnesia following two injections of MDMA has been reported in rats in the Morris Water Maze and the object recognition paradigms (Camarasa et al., 2008). Acute administration of MDMA before acquisition trial of the PA test was observed to decrease retention 24 h later (Barrionuevo et al., 2000). However, our study is in agreement with results obtained by Trigo and colleagues. They showed that MDMA administered acutely improved the performance of an active avoidance task in mice in a dose-dependent manner (Trigo et al., 2008). In our previous studies, a tendency toward a procognitive effects was noticed only after administration of the dose 2.5 and 5 of MDMA whereas the doses 1 and 10 mg/kg did not influence cognitive processes (Budzynska et al., 2017). Moreover, it is worth to mention that our previous experiments showed administration of nicotine at the dose of 0.05 mg/kg had no effect on memory consolidation. Whereas higher dose of nicotine (0.5 mg/kg) enhanced the cognitive processes in the animals. Present studies show that animals treated with subthreshold doses of nicotine and MDMA presented a marked procognitive effect. This action may be due to the interaction of drugs-induced nAChRs activation, because both nicotine and MDMA activate these receptors (Ciudad-Roberts et al., 2013). MDMA acts as a partial agonist of α7 nAChR, and as an antagonist on α4β2 nAChR (Garcia-Ratés et al., 2010), whereas nicotine activates both α7 and α4β2 types of nAChRs in the brain. Moreover, Bancroft and Levin (2000) showed that chronic nicotine treatment reversed cognitive dysfunction induced by the blockade of α4β2 subtype of nAChR in the ventral hippocampus. Thus, our results may suggest the existence of interactions between MDMA and nicotine on cholinergic systems in the brain. As we did not detect significant changes in the protein level of α7 nAChR in the prefrontal cortex nor in the hippocampus after MDMA and nicotine administration we may suggest that observed effect resulted from receptor activation rather than from its expression regulation. It is worth mentioning that, molecular changes such as receptor expression typically occurs after chronic administration (Thomsen and Mikkelsen, 2012), thus further studies are needed.

Furthermore, it has been shown that the hippocampus and the neocortex are the structures involved in memory processes and lesions of these brain structures impaired learning and memory in laboratory animals (Clarke and Adermark, 2010). The hippocampus plays a pivotal role in contextual fear conditioning, spatial and emotional learning and working memory (Jeltsch et al., 2004; Calandreau et al., 2007; Elvander-Tottie et al., 2009). Due to the fact that the hippocampus and the cortex are involved in cognitive processes (Bird and Burgess, 2008) we have measured processes connected with oxidative stress after co-administration of nicotine and MDMA in these structures as well as in the whole brain.

Oxidative stress has been implicated in cognitive impairments in dementia, mild cognitive impairment (MCI) as well as Alzheimer's disease (AD) (Newton et al., 2015). In animal models, scopolamine-induced memory disorders have been associated with increased oxidative stress in the whole brain as well as in specific structures (i.e. hippocampus and prefrontal cortex that are involved in memory and learning). Furthermore, scopolamine has been found to reduce concentration of low molecular weight intracellular antioxidants, mainly glutathione, and therefore to further boost damage of CNS cells through increased lipid peroxidation (Budzynska et al., 2013). Additionally, cognitive deficits have also been related to oxidative stress in the process of aging brain tissues and to the neurodegenerative processes that result from the vulnerability of the CNS to damage caused by overproduction of ROS (Kapogiannis and Mattson, 2011). Therefore, oxidative stress may be one of the factors influencing the disturbance of cognitive functions.

Club drugs improve cognitive functions by increasing the concentration of neurotransmitters; however, they also cause increased production of free radicals. MDMA, like other amphetamine derivatives, shows multi-directional pro-oxidative action. Firstly, it disturbs cellular energy metabolism through intensification of the mitochondrial electron transport chain (ETC)-main source of ROS. MDMA, causing hyperthermia (Kiyatkin et al., 2014), changes the efficiency of ETC, which in physiological conditions drops to about 40% (i.e. only 40% of energy is stored in form of ATP while the rest is liberated in form of heat). Such prolonged positive energy balance and intensification of ETC course with simultaneous increase in blood pressure and heart rate caused by MDMA (Schindler et al., 2014) causes increased oxygen consumption by the whole body, especially by the brain. As CNS uses the most oxygen, the percentage of ROS production is also the highest there. Secondly, free radicals are produced during auto-oxidation of neurotransmitters (mainly dopamine) (Capela et al., 2009), the concentration of which is significantly increased by MDMA (Monks et al., 2004). And finally, ROS may also be produced in the course of redox reaction in the metabolic processes of the drug molecule (de la Torre et al., 2004). An additional mechanism by which a combination of nicotine and MDMA could result in increased ROS production after an acute dose was reported by (Garcia-Ratés et al., 2010) by which these drugs could synergistically activate α7 nAChRs These receptors have great permeability for calcium ions and an increase of the cytosolic calcium level activates calcium-dependent pathways involved in the generation of ROS (Garcia-Ratés et al., 2010).

Our study confirmed pro-oxidative, dose-dependent action of MDMA. Through impairment in activities of antioxidant enzymes and decrease in TAS level it caused increase in lipids peroxidation level expressed as an elevated concentration of MDMA. In our experiment, the structure, which was the most significantly affected by MDMA intoxication, was the hippocampus, although the marked changes were also noted in the whole brain. Although we revealed the pro-oxidative effects of MDMA and nicotine, the short experimental period did not show any negative influence of these drugs on cognitive processes. Thus we may suggest that observed procognitive effects may result rather from activation of nAChRs then pro-oxidative action. The further aim of the present study was to evaluate the effects of the co-administration of the MDMA on nicotine-induced locomotor sensitization. The present results confirmed that repeated daily injections of nicotine induced increases in the locomotor activity in mice, most strongly expressed after nicotine challenge. Our present experiments showed that MDMA injection increased locomotor activity in nicotine-experienced mice when compared with both the first pairing day and the response to acute MDMA challenge in animals pre-exposed to saline.

Locomotor sensitization may indicate a gradual and sustained increase in psychomotor activating effects of drugs (e.g. psychostimulants). This effect occurs after repeated and intermittent administration of drugs of abuse. Neuroplasticity in the areas of the brain associated with sensitization may be a pivotal mechanism in the development of drug addiction. Furthermore, behavioral sensitization may be an important factor in the transition from casual use to compulsive drug seeking and neuroplasticity in the areas of the brain associated with sensitization may be a key mechanism in the development of drug addiction (Robinson and Berridge, 2008). The mesolimbic dopaminergic system, has been shown to play a crucial role in drug-induced sensitization of hyperlocomotion, reinforcement, reward, and some acute effects of drugs of abuse (Heidbreder and Thompson, 1996). Since most drugs of abuse activate dopaminergic neurotransmission polydrug abuse may intensify this effect (Vanderschuren and Kalivas, 2000). Moreover, the previous studies have found that repeated intermittent administration of nicotine induces locomotor sensitization (Biala and Staniak, 2010). Additionally, our study revealed that a nonactive by itself dose of MDMA (5 mg/kg) can enhance the expression of sensitization to nicotine but the mechanisms have not been explained. Although, literature reports activity of this dose (Varela et al., 2011) in our study it was chosen on the basis of our preliminary experiments. Locomotor activity after administration of MDMA at the dose of 5 mg/kg in our laboratory conditions was not statistically significant in comparison with control group. It may depend on the conditions in the laboratory or strain of the mice as the lack of influence of acute injection of MDMA at the dose of 8 mg/kg in mice reported by Maldonado and Navarro (2000).

MDMA is well known to increase dopaminergic neurotransmission through blocking DA reuptake and increasing release in the brain (Brennan et al., 2009). It was shown that nicotine also can increase DA level through AChRs, activation of nAChRs by nicotine, indirectly increases the release of DA in the reward system (Dani and De Biasi, 2001). Our biochemical studies on biomarkers of oxidative stress proved, that co-administration of MDMA with nicotine additionally increased the oxidative stress level within mice brains, and here the structure which was more affected by those two drugs was the cortex. Additionally, as antagonists of α4bβ2, but not α7 nAChR block the hyperlocomotor (acute and sensitized) or rewarding effects of nicotine it was revealed that these receptors are pivotal for aforementioned functions. (Rahman et al., 2007). Furthermore, other experimental data has suggested that heteromeric nicotinic receptors are involved in locomotor sensitization induced by MDMA, whereas the influence of α7 nicotinic receptors was excluded (Ciudad-Roberts et al., 2013). Therefore, we may hypothesize a synergistic effect of nicotine and MDMA on the activation of central dopaminergic and cholinergic functions.

To conclude, the present experiments demonstrated that co-administration of MDMA and nicotine improved consolidation of memory processes. Furthermore, one of the main findings of the present study is the existence of influence of MDMA on locomotor activity in nicotine-sensitized mice. The data indicate that common neural systems are impacted by exposure to nicotine and MDMA. Although our study proves pro-oxidative effects of MDMA and nicotine, the short experimental period did not show any negative influence of these drugs on cognitive processes. However, as our results indicate the increase of oxidative stress after administration of MDMA and nicotine, long term use or application of high doses may induce neurodegeneration and neural defects causing cognitive deficits. Both drugs are often used in combination, especially by young people, thus these results may help explaining why psychoactive substances are being co-abused and why this polydrug administration is still a social problem.

## Author contributions

BB: planned study and performed behavioral experiments; AB-C: performed biochemical study; AW: performed receptor study; BB, AW, and AB-C: analyzed data; BB, AB-C, KK, AW, JK, MK-S, and GB: prepared the manuscript.

### Conflict of interest statement

The authors declare that the research was conducted in the absence of any commercial or financial relationships that could be construed as a potential conflict of interest.

## Footnotes

^1^http://www.unodc.org/unodc/en/data-and-analysis/WDR-2010.html">http://www.unodc.org/unodc/en/data-and-analysis/WDR-2010.html, 2010
