# Supplementary material for: Acute MDMA and Nicotine Co-administration: Behavioral Effects and Oxidative Stress Processes in Mice
Source: Front Behav Neurosci. 2018 Aug 2;12:149. doi: 10.3389/fnbeh.2018.00149 (PMC6082960; doi:10.3389/fnbeh.2018.00149)
Supplement: Supplementary file 1 [file Data_Sheet_1.docx]

Acute MDMA and nicotine co-administration: behavioral effects and oxidative stress processes in mice

Supplementary data

Barbara Budzynska^1^*, Artur Wnorowski^2^, Katarzyna Kaszubska^1^, Grazyna Biala^1^,

Marta Kruk-Słomka^1^, Jacek Kurzepa^3^ and Anna Boguszewska-Czubara^3^

^1^ Department of Pharmacology and Pharmacodynamics, Medical University of Lublin, Lublin, Poland

^2^ Department of Biopharmacy, Medical University of Lublin, Lublin, Poland

^3^ Department of Medical Chemistry, Medical University of Lublin, Lublin, Poland

# Materials and methods

## Chemicals

Antibodies raised against α7 nAChR (ab23832) and β-actin (ab6276) were obtained from Abcam (Cambridge, UK). Horseradish peroxidase (HRP)-conjugated secondary antibodies were bought from Santa Cruz Biotechnology (Dallas, TX, USA).

## Immunoblotting

The animals administrated with nicotine (0.05 mg/kg), MDMA (1 mg/kg), combination of nicotine and MDMA or with vehicle were decapitated, and the whole brain from each animal was carefully taken out and rinsed in isotonic saline solution to remove residual blood. The prefrontal cortexes and the hippocampi were rapidly dissected and frozen at -80°C.

Prefrontal cortexes and hippocampal samples were lysed in Cell Lysis Buffer supplemented with 1 mM PMSF as well as protease and phosphatase inhibitor cocktail. The BCA assay was employed to measure the protein concentration of each lysate. Equivalent amount of proteins was then loaded on polyacrylamide gels, separated by polyacrylamide gel electrophoresis under reducing conditions, and transferred onto PVDF membranes. After blocking the membranes with 3% milk in Tris-buffered saline/0.1% Tween-20, the blots were incubated with the anti-α7 nAChR or anti-β-actin primary antibody, followed by incubation with a HRP-conjugated secondary antibody. The immunoreactive protein bands were visualized by chemiluminescence, and quantified by the means of volume densitometry with Fiji image processing package (Schindelin et al., 2012) using β-actin as loading control.

# Results

## Expression of α7 nAChR in prefrontal cortex and hippocampus upon MDMA and nicotine treatment

Homomeric α7 nAChR is involved in the generation of MDMA-dependent neurotoxicity. In order to evaluate whether the MDMA can affect the expression of this receptor we monitored the expression levels of α7 nAChR in prefrontal cortex and hippocampus of mice treated with MDMA alone or in combination with nicotine. Expression of the receptor was measured by western blotting using previously validated anti-α7 nAChR antibody (Mielke and Mealing, 2009). We detected no significant changes in the expression level of α7 nAChR in prefrontal cortex nor in hippocampus of tested animals (Fig. 1).

# Reference

1. Schindelin, J., Arganda-Carreras, I., Frise, E., Kaynig, V., Longair, M., Pietzsch, T., et al. (2012). Fiji: an open-source platform for biological-image analysis. Nat. Methods. 9, 676-682.
2. Mielke, J.G., Mealing, G.A. (2009). Cellular distribution of the nicotinic acetylcholine receptor alpha7 subunit in rat hippocampus. Neurosci. Res. 65, 296-306.


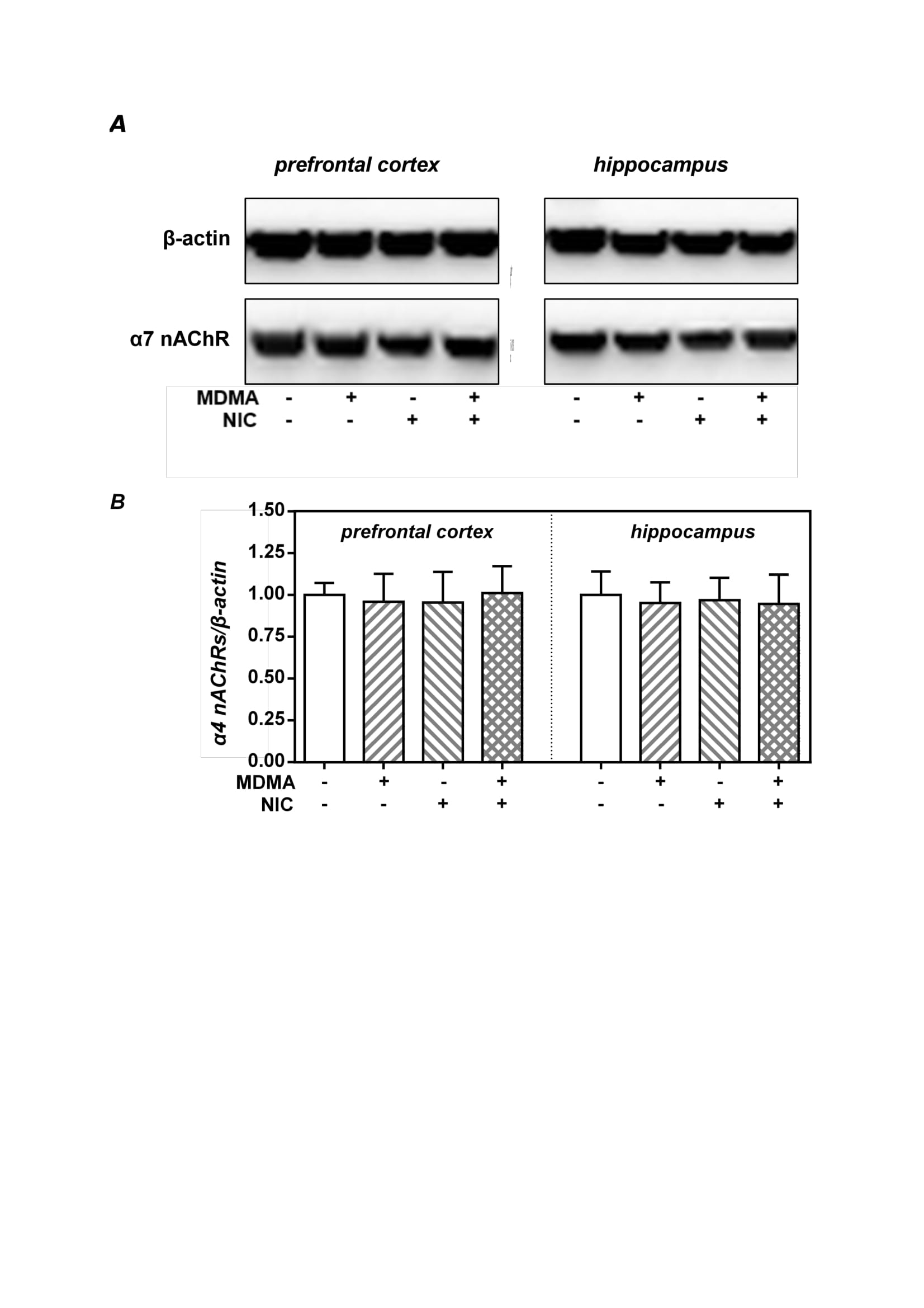
**Figure 1.** Expression of α7 nAChR in response to MDMA and nicotine. Mice were treated with vehicle, nicotine (0.05 mg/kg), MDMA (1 mg/kg) or the combination of nicotine (0.05 mg/kg) and MDMA (1 mg/kg). The prefrontal cortex and the hippocampus were collected from each animal 30 min post treatment and lysed. Obtained lysates were electrophoretically separated and blotted using high specificity antibodies recognizing murine α7 nAChR. β-actin was used as a loading control. (A) Representative blots. (B) Densitometric quantification of immunoreactive bands. Values on the graph represent means ± SD from 8 samples per treatment group. Two-way ANOVA followed by Tukey's post-hoc test revealed no statistically significant differences in mean expression levels of α7 nAChR across treatment groups in the prefrontal cortex nor in the hippocampus.
